# The concordance of directly and indirectly measured built environment attributes and physical activity adoption

**DOI:** 10.1186/1479-5868-8-72

**Published:** 2011-07-07

**Authors:** Kristen M McAlexander, Scherezade K Mama, Ashley Medina, Daniel P O'Connor, Rebecca E Lee

**Affiliations:** 1 Department of Applied Physiology and Wellness, Southern Methodist University, Dallas, TX, USA; 2 Texas Obesity Research Center, Department of Health and Human Performance, University of Houston, Houston, TX, USA

## Abstract

**Background**

Physical activity (PA) adoption is essential for obesity prevention and control, yet ethnic minority women report lower levels of PA and are at higher risk for obesity and its comorbidities compared to Caucasians. Epidemiological studies and ecologic models of health behavior suggest that built environmental factors are associated with health behaviors like PA, but few studies have examined the association between built environment attribute concordance and PA, and no known studies have examined attribute concordance and PA adoption.

**Purpose**

The purpose of this study was to associate the degree of concordance between directly and indirectly measured built environment attributes with changes in PA over time among African American and Hispanic Latina women participating in a PA intervention.

**Method**

Women (*N *= 410) completed measures of PA at Time 1 (T1) and Time 2 (T2); environmental data collected at T1 were used to compute concordance between directly and indirectly measured built environment attributes. The association between changes in PA and the degree of concordance between each directly and indirectly measured environmental attribute was assessed using repeated measures analyses.

**Results**

There were no significant associations between built environment attribute concordance values and change in self-reported or objectively measured PA. Self-reported PA significantly increased over time (*F*(1,184) = 7.82, *p *= .006), but this increase did not vary by ethnicity or any built environment attribute concordance variable.

**Conclusions**

Built environment attribute concordance may not be associated with PA changes over time among minority women. In an effort to promote PA, investigators should clarify specific built environment attributes that are important for PA adoption and whether accurate perceptions of these attributes are necessary, particularly among the vulnerable population of minority women.

## Background

Ethnic minority women report lower levels of physical activity (PA) [1] and are at higher risk for obesity and its comorbidities compared to Caucasian women [2,3]. Further, health attitudes and behaviors can differ by ethnicity [4-6]. Studies that investigate built environment measurement factors related to the adoption of PA are extremely important since consistent evidence suggests that neighborhood characteristics and health behaviors are significantly related [7-10]. Research suggests that factors influencing PA adoption are different for men and women [11,12], and there may be different factors influencing behavior adoption versus maintenance [13,14].

Ecologic models of human behavior have evolved over decades in the fields of sociology, psychology and public health [7,15-17], and their significance to PA is now widely recognized [7,16-18]. Neighborhood built environment changes can benefit all people in a surrounding neighborhood rather than only focusing on changing individual behavior [17]. These changes can include building and improving physical activity resources (PARs), sidewalks and bicycle facilities (e.g., bicycle lanes, bicycle route signs), and can be more permanent than interventions focusing on individual-level changes. Specific built environment attributes can provide opportunities, support, and cues to help people adopt PA and may complement individual-level programs. Empirical evidence consistently supports these associations [7-10], but less is known about how built environment attributes affect PA adoption, especially among vulnerable populations like women [17] for whom predictors of the adoption and maintenance of PA can differ [19].

The concordance of directly measured built environment attributes and indirectly measured built environment attributes has been significantly associated with PA [20,21]. Concordance is measured by the strength and direction of the correlation between directly measured and indirectly measured variables of the built environment [20,22]. Direct built environment measures may provide objective data, unbiased by resident perceptions, as well as specific evidence for policy change impacting urban planning and transportation. Indirect built environment measures include self-reported data on perceived environmental attributes and can provide insight on individual attitudes about the built environment. Both direct and indirect measures of the built environment have been associated with PA separately [10,23-26], but few studies have examined the association between concordance and PA [20,21,27]. Gebel and colleagues found a fair overall agreement between objectively determined walkability and perceived walkability, but adults with lower educational attainment and lower incomes or who were overweight were more likely to misperceive their high walkable neighborhood as low walkable [20]. Findings suggest the potential for PA promotion and persuasion strategies to address non-concordance [20], but these associations have not been examined for PA adoption or among minority women. Individuals who are less physically active may also be more likely to misperceive their built environment compared to those who are more physically active [20,27], suggesting that the concordance of direct and indirect built environment measurement may be dynamic and related to PA and/or PA adoption.

The purpose of this study was to measure the associations between built environment attribute concordance and PA adoption among African American and Hispanic or Latina women. We hypothesized that women who demonstrated a stronger concordance between directly and indirectly measured built environment attributes would exhibit increased PA over time or PA adoption.

## Methods

The current study is a secondary analysis using data from the Health is Power (HIP) project. Originating in 2005, the HIP project was a five-year, longitudinal study funded by the National Cancer Institute of the National Institutes of Health (R01 CA109403) to increase PA and improve dietary habits in African American and Hispanic or Latina women in Houston and Austin, Texas. The HIP project was approved by the Committee for the Protection of Human Subjects at the University of Houston, and participants provided written informed consent to participate. The investigators certified that all applicable institutional and governmental regulations concerning the ethical use of human research volunteers were followed during the investigation.

### Study Design

Environmental cross-sectional data and longitudinal individual data were used to measure the association between concordance between directly and indirectly measured built environment attribute data and changes in PA over time among African American and Hispanic or Latina women.

### Participants

Four hundred ten African American and Hispanic or Latina women (311 in Houston and 99 in Austin) were enrolled in the study. Of those enrolled in Houston, 84.6% identified as African American and 15.4% identified as Hispanic or Latina; all participants in Austin identified as Hispanic or Latina [28].

### Measures

#### Individual Measures

Sociodemographic measures of age, gender, marital status, employment status, years of education, and income range were measured using the Maternal and Infant Health Assessment (MIHA) [29]. Modeled on the Center for Disease Control's (CDC) Pregnancy Risk Assessment Monitoring System (PRAMS), the MIHA includes items that have been used with samples representing a diverse range of ethnicities and socioeconomic status categories [29,30].

To assess self-reported PA levels, the International Physical Activity Questionnaire (IPAQ) Long Form was used. Median values and interquartile ranges were computed for walking, moderate-intensity activities, vigorous-intensity activities and for a combined total PA score. The total PA score at Time 1 (T1) was used along with the total PA score at Time 2 (T2) to measure changes, or differences, in PA from T1 to T2. All continuous scores were expressed in MET-minutes, computed by multiplying the MET score of an activity by the minutes performed [31].

Accelerometers (MTI Actigraph) were used to objectively assess the amount and intensity of PA participants did each day [32]. Participants wore accelerometers for seven consecutive days at to assess typical PA for moderate-intensity or greater activity. Days with eight or more valid hours of data, or fewer than 30 consecutive zero counts, were included in analyses [32]. A daily average of the amount of moderate-vigorous accelerometer-measured PA (MVPA) at T1 and T2 was used to measure changes in PA from T1 to T2, or PA adoption.

Body Mass Index (BMI = kg/m^2^) and percent body fat were used as measures of body composition. Participants removed shoes and heavy outer clothing, and trained research assistants measured height using a portable stadiometer (Seca 225 Hite Mobile Measuring Device; North Bend, Washington) and weight using a bioelectrical impedance monitor with scales (The TBF-310 & the TBF-300; Tanita Corporation, Chicago of America, Arlington Heights, IL). Percent body fat was measured using the Tanita integrated bioelectrical impedance body fat monitor and scale (Tanita Body Fat Analyzer, TBF 105, Tanita Corporation of America, Inc., Arlington Heights, IL).

### Procedures

#### Individual Assessments

Women were recruited to the HIP project via the media, brochures, churches and internet communication over the course of one year. Interested participants were invited to call the HIP project and complete a telephone screener. Women were screened to meet the following inclusion criteria: (1) self identified as African American or Hispanic or Latina, (2) between the ages of 25 and 60 years old, to include adults outside the college age range, (3) able to read, speak, and write in English or Spanish, (4) not pregnant or planning to become pregnant within the next 12 months, (5) a Harris or Travis County resident, (6) not planning on moving in the next 12 months, (7) physically inactive or doing fewer than 30 minutes of physical activity per day on 3 or more days per week, and (8) able to pass the Physical Activity Readiness Questionnaire (PAR-Q) [33]. Eligible participants completed an interviewer administered self-report environmental perception questionnaire at T1 and self-reported PA measures at T1 and T2. Participants also completed a seven day accelerometer protocol at T1 and T2 and were compensated for completing assessments at each time point [32].

#### Neighborhood Assessments and GIS Development

As reported previously [34], participant street addresses were geocoded and plotted by a trained Geographical Information Systems (GIS) specialist using ArcGIS software [35]. Each participant's neighborhood was restricted to an 800 meter or approximately 1/2 mile radius buffer. Environmental assessments were completed during the intervention to capture neighborhoods at the same time in order to avoid simultaneity bias [36]. In order to compare directly measured PAR accessibility to indirectly measured PAR accessibility, the total number of accessible PARs was calculated for each participant's neighbourhood using the Physical Activity Resource Assessment instrument [37-39]. Path maintenance was assessed based on the amount of debris and/or the overall condition of the facility, and pedestrian and bicycle facility density was calculated by counting the number of pedestrian and bicycle facilities within each predefined neighborhood (i.e. 800 m radius circle) using the Pedestrian Environment Data Scan instrument [35,40].

### Statistical Analyses

Descriptive analyses were completed to examine the frequency and distribution of individual and environmental variables. BMI, body fat percentage, self-reported PA and accelerometry were analyzed at T1 and T2, and bivariate analyses were conducted among all variables, including directly measured and indirectly measured built environment variables, BMI, body fat percentage, self-reported PA, accelerometer measured PA, sociodemographic variables and ethnicity.

Repeated measures analyses were conducted to determine if concordance values were associated with PA adoption or PA changes from T1 to T2, for both the IPAQ and accelerometer measured PA. Because bivariate and model-based analyses suggested no significant associations among any individual and built environment variables, only ethnicity was included in the repeated measures analyses in order to examine differences among African Americans and Hispanic or Latinas. Interaction terms were considered in the models, and the F-ratio test significance was set at *p *< .05. All statistical analyses were conducted in SPSS Version 18.0 (SPSS 18.0 for Windows; SPSS Inc, Chicago, Ill).

## Results

### Descriptive Characteristics

Participants (*N *= 410) were mostly obese (T1 *M *BMI = 34.5 kg/m^2^, *SD *= 7.9; T2 *M *BMI = 34.2 kg/m^2^, *SD *= 8.1), highly educated (89% completed college or completed some college) and nearly half reported an income over 400% of the Federal Poverty Level for a family of four in 2007 [41]. African American women (*M *= 3326.5 MET minutes per week, *SD *= 3169.5 and *M *= 24.4 minutes MVPA per day, *SD *= 19.9) were more physically active than Hispanic or Latina women (*M *= 2840.5 MET minutes per week, *SD *= 2067.0 and *M *= 11.7 minutes MVPA per day, *SD *= 9.1) according to self-reported and objectively-measured PA assessments [28]. Ethnicity, BMI, percent body fat and PA were not significantly associated with any built environment attribute. All descriptive individual and environmental data have been reported previously [28,34].

### Built Environment Attribute Concordance and PA Adoption

Repeated measures analyses revealed no significant relationships between any built environment attribute concordance value and PA adoption or PA changes from T1 to T2 for total self-reported or objectively measured PA. Self-reported PA significantly increased over time (*F*(1,184) = 7.82, *p *= .006) [28] but did not significantly vary by ethnicity, BMI, percent body fat and directly or indirectly measured built environment attributes. Objectively measured PA did not significantly increase over time [28]. Repeated measures analyses results are presented in Tables 1 and 2.

**Table 1:** Repeated Measures results for self-reported PA adoption

Built Environment Concordance Attribute Used	Effect	*df*	*F*	*p*-value
*PAR Access*				
	Time	1	2.69	.10
	Time*Ethnicity	1	.00	.99
	Time*PAR Access Concordance	1	.12	.73
	Time*Ethnicity*PAR Access Concordance	1	.71	.40
	Error	162		
*Path Maintenance*				
	Time	1	2.39	.12
	Time*Ethnicity	1	.01	.91
	Time*Path Maintenance Concordance	1	.84	.36
	Time*Ethnicity*Path Maintenance Concordance	1	.01	.91
	Error	153		
*Pedestrian Facility Density*				
	Time	1	2.17	.14
	Time*Ethnicity	1	.21	.65
	Time*Pedestrian Facility Density Concordance	1	.16	.69
	Time*Ethnicity*Pedestrian Facility Density Concordance	1	.47	.49
	Error	158		
*Bicycle Facility Density*				
	Time	1	7.22	.01
	Time*Ethnicity	1	.61	.44
	Time*Bicycle Facility Density Concordance	1	.79	.38
	Time*Ethnicity*Bicycle Facility Density Concordance	1	.44	.51
	Error	173		

**Table 2:** Repeated measures results for objectively-measured PA adoption

Built Environment Concordance Attribute Used	Effect	*df*	*F*	*p*-value
*PAR Access*				
	Time	1	1.85	.18
	Time*Ethnicity	1	.61	.44
	Time*PAR Access Concordance	1	.20	.66
	Time*Ethnicity*PAR Access Concordance	1	1.83	.19
	Error	36		
*Path Maintenance*				
	Time	1	.80	.38
	Time*Ethnicity	1	.29	.59
	Time*Path Maintenance Concordance	1	.35	.56
	Time*Ethnicity*Path Maintenance Concordance	1	.22	.64
	Error	35		
*Pedestrian Facility Density*				
	Time	1	1.63	.21
	Time*Ethnicity	1	.60	.45
	Time*Pedestrian Facility Density Concordance	1	.86	
	Time*Ethnicity*Pedestrian Facility Density Concordance	1	.94	.36
	Error	36		
*Bicycle Facility Density*				
	Time	1	.118	.73
	Time*Ethnicity	1	.07	.80
	Time*Bicycle Facility Density Concordance	1	.23	.63
	Time*Ethnicity*Bicycle Facility Density Concordance	1	.09	.77
	Error	40		

## Discussion

We hypothesized that for our sample of minority women, a stronger concordance of directly and indirectly measured built environment attributes would be significantly associated with PA adoption. Objectively measured PA did not significantly increase, but self-reported PA did significantly increase from T1 to T2. PA changes over time did not vary by ethnicity or any concordance measure.

No earlier study has measured the association between built environment attribute concordance and PA changes over time, but PA has been reported to be a significant correlate of built environment attribute concordance [20]. In particular, one study found lower concordance among women with lower income, PA and self-efficacy for PA [27]. Also, other findings suggest that indirectly measured neighborhood data are more closely linked to self-reported PA than directly measured neighborhood data [27,42]. Unlike studies measuring direct and indirect built environment attribute concordance, our sample consisted solely of minority women. The relationships between PA and attribute concordance might differ for our population, as earlier findings suggest that the degree of built environment non-concordance can vary among certain population subgroups [27]. Also, our samples were of high SES, particularly for income and education; we also assessed a wider variety of neighborhood types than previous studies [20,42], increasing the generalizability of our findings.

Although not all of our participants exhibited increased PA over time or PA adoption, this study initiates an evidence base where no similar data exist. PA adoption is an essential component to a healthy lifestyle [3,43], yet no known study has measured the associations of PA changes over time with built environment concordance values. Further, this study investigated these relationships among minority women. Although African American and Hispanic or Latina women continue to be disproportionately physically inactive compared to white women [2,3], they continue to be understudied in the built environment literature [17].

Other strengths of this study include the use of a self-reported PA questionnaire and accelerometry to measure PA changes over time, providing a comprehensive assessment of PA. Although similar studies have been cross-sectional in nature [20,27,44], our study measured PA longitudinally. We also used measured BMI and body fat percentage, rather than self-report, helping to reduce bias and measurement error.

Our study is not without limitations. Due to adherence, cost and logistic reasons, the number of participants who wore accelerometers was lower than those who completed the self-reported PA questionnaire at T1 and T2. Resources are needed for future studies to recruit and assess an equal number of participants for multiple PA measures to provide a more comprehensive PA assessment. McCormack and colleagues found that residents' perceived behavior control cognitions were mediators in the relationship between the built environment and PA [45], and future work is needed to include additional individual-level variables that might help explain the variability of attribute perception(s) and PA changes among these populations.

This study investigated built environment measurement concordance and PA changes over time among minority women. Inaccurate perceptions of built environment attributes were *not *associated with PA level change. Future PA interventions and supportive communities could promote built environment attributes (e.g., park amenities, clean baseball fields, long walking trails) in an attempt to increase PA. Policies could attempt to increase facility and street signage in an effort to promote PA, particularly among ethnically diverse neighborhoods.

## Conclusions

Although the influence of the built environment on individual health behaviors has been well established, more study of the interactions between specific built environment attributes and intra-individual factors like gender and ethnicity is needed. These linkages are not well understood, and the applicability of ecological frameworks could be limited if the relationships between built environment attributes and health behaviors vary for certain personal characteristics. In an effort to promote PA, investigators should clarify specific built environment attributes that are important for PA adoption and whether accurate perceptions of these attributes are necessary, particularly among the vulnerable population of minority women.

## List of abbreviations

BMI: Body Mass Index; CDC: Center for Disease Control and Prevention; GIS: Geographical Information Systems; HIP: Health is Power; IPAQ: International Physical Activity Questionnaire; MIHA: Maternal and Infant Health Assessment; MVPA: Moderate and Vigorous Physical Activity; PA: Physical Activity; PAR: Physical Activity Resource; PARA: Physical Activity Resource Assessment Instrument; PAR-Q: Physical Activity Readiness Questionnaire; PEDS: Pedestrian Environment Data Scan; PRAMS: Pregnancy Risk Assessment Monitoring System; SES: Socioeconomic Status; T1: Time 1; T2: Time 2.

## Competing interests

The authors declare that they have no competing interests.

## Authors' contributions

KMM primarily wrote the manuscript. SKM helped to coordinate the study and assisted with data collection. AM provided geographic data support and also helped with data collection. DPO assisted with analyses and interpretation of data. REL conceived the original study, secured funding, provided individual and environmental data and intensive guidance through all phases of the manuscript. All authors read and approved the final manuscript.
